# Multiplex PCR for the Identification of Pathogenic *Listeria* in *Flammulina velutipes* Plant Based on Novel Specific Targets Revealed by Pan-Genome Analysis

**DOI:** 10.3389/fmicb.2020.634255

**Published:** 2021-01-15

**Authors:** Fan Li, Qinghua Ye, Moutong Chen, Jumei Zhang, Liang Xue, Juan Wang, Shi Wu, Haiyan Zeng, Qihui Gu, Youxiong Zhang, Xianhu Wei, Yu Ding, Qingping Wu

**Affiliations:** ^1^Guangdong Provincial Key Laboratory of Microbial Safety and Health, State Key Laboratory of Applied Microbiology Southern China, Guangdong Institute of Microbiology, Guangdong Academy of Sciences, Guangzhou, China; ^2^School of Biology and Biological Engineering, South China University of Technology, Guangzhou, China; ^3^College of Food Science, South China Agricultural University, Guangzhou, China; ^4^Department of Food Science and Technology, Jinan University, Guangzhou, China

**Keywords:** novel target gene, *Listeria*, pan-genome analysis, multiplex PCR, mushroom

## Abstract

*Listeria* spp. is an important foodborne disease agent, often found in the fresh mushroom (*Flammulina velutipes*) and its production environment. The aim of this study was to develop multiplex PCR for rapid identification of *Listeria monocytogenes* and *Listeria ivanovii*, and nonpathogenic *Listeria* in *F. velutipes* plants. Pan-genome analysis was first used to identify five novel *Listeria*-specific targets: one for the *Listeria* genus, one for *L. monocytogenes*, and three for *L. ivanovii*. Primers for the novel targets were highly specific in individual reactions. The detection limits were 10^3^–10^4^ CFU/mL, meeting the requirements of molecular detection. A mPCR assay for the identification of pathogenic *Listeria*, with primers targeting the novel genes specific for *Listeria* genus (*LMOSLCC2755_0944*), *L. monocytogenes* (*LMOSLCC2755_0090*), and *L. ivanovii* (*queT_1*) was then designed. The assay specificity was robustly verified by analyzing nonpathogenic *Listeria* and non-*Listeria* spp. strains. The determined detection limits were 2.0 × 10^3^ CFU/mL for *L. monocytogene*s and 3.4 × 10^3^ CFU/mL for *L. ivanovii*, for pure culture analysis. Further, the assay detected 7.6 × 10^4^ to 7.6 × 10^0^ CFU/10 g of pathogenic *Listeria* spiked into *F. velutipes* samples following 4–12 h enrichment. The assay feasibility was evaluated by comparing with a traditional culture-based method, by analyzing 129 samples collected from different *F. velutipes* plants. The prevalence of *Listeria* spp. and *L. monocytogenes* was 58.1% and 41.1%, respectively. The calculated κ factors for *Listeria* spp., *L. monocytogenes*, and *L. ivanovii* were 0.97, 0.97, and 1, respectively. The results of the novel mPCR assay were highly consistent with those of the culture-based method. The new assay thus will allow rapid, specific, and accurate detection and monitoring of pathogenic *Listeria* in food and its production environment.

## Introduction

*Listeria* is a genus of gram-positive facultative anaerobic bacilli that is widely distributed in food and natural environments. The genus *Listeria* includes many species: *Listeria monocytogenes*, *Listeria innocua*, *Listeria ivanovii*, *Listeria seeligeri*, *Listeria welshimeri*, *Listeria grayi*, *Listeria rocourtiae*, *Listeria fleischmannii*, *and Listeria marthii*, etc. (Vitullo et al., 2013). Among them, *L. monocytogenes* and *L. ivanovii* are human and animal pathogens (Snapir et al., 2006; Jagadeesan et al., 2019; Lee et al., 2020). These bacteria can cause severe listeriosis, leading to spontaneous abortion, neonatal sepsis, and meningoencephalitis, and the post-infection mortality rate ranges from 20 to 30% (Radoshevich and Cossart, 2018; Ranjbar and Halaji, 2018), which places this pathogen among the most frequent causes of death from foodborne illnesses (Tamburro et al., 2015b).

*Listeria* spp. can contaminate food at multiple stages of production and distribution because it is ubiquitous in the environment and tolerates harsh environmental conditions (Tamburro et al., 2015a). Edible mushrooms are potential vehicles for the transmission of pathogenic *Listeria* (Chen et al., 2014, 2018). For example, our previous study found that the prevalence of *Listeria* spp. and *L. monocytogenes* in *F. velutipes* production plants is 52.5% (155/295) and 18.6% (55/295), respectively (Chen et al., 2014). However, currently, the gold standard method for detection of *Listeria* in *F. velutipes* is the conventional culture method (Chen et al., 2014; Tao et al., 2017), which is labor-intensive, expensive, and time-consuming. It is therefore important to develop rapid and accurate diagnostic techniques or tools for the detection and distinguishing of pathogenic *Listeria* in *F. velutipes*, to facilitate introduction of appropriate intervention measures in the *F. velutipes* production chain to reduce the risk of *Listeria* contamination.

Polymerase chain reaction (PCR) has been widely employed as a rapid and specific method for the detection of *Listeria* in a variety of foods and processing environments because of its high specificity, sensitivity, time-saving and easy operation (Norton et al., 2000; Ryu et al., 2013; Rosimin et al., 2016; Sheng et al., 2018). However, most of the reported PCR-based methods for the identification and characterization of *Listeria* target the bacterial virulence genes (Doijad et al., 2011; Mao et al., 2016; Li et al., 2017; Tamburro et al., 2018, 2019), or 16S and 23S rRNA genes (Czajka et al., 1993; Hellberg et al., 2013), which have a limited number of targets, and some genes cannot accurately identify target bacteria. For example, avirulent *L. monocytogenes* strains may lack one or more virulence genes due to mutations or low gene expression under certain conditions (Nishibori et al., 1995). In addition, the highly conserved sequence of the 16S rRNA gene may fail to distinguish between *Listeria* species, especially *L. monocytogenes*, *L. innocua*, and *L. welshimeri* (Hellberg et al., 2013; Tao et al., 2017). Therefore, mining of target genes with high species-specificity is vital for improved accuracy, specificity, and efficiency of pathogen detection.

At present (as of 27 May 2019), a large number of sequenced genomes of *Listeria* strains are available from the Genome Database of the National Center for Biotechnology Information (NCBI)^1^. Using a wealth of genome data, pan-genome analysis has become a representative discipline for studying an entire repertoire of gene families in genomes of a clade of pathogenic bacteria, which provides not only the whole set of genes shared among *Listeria* species but also can be applied for inter-species differentiation analysis to mine species-specific genes (Kuenne et al., 2013; Kim et al., 2020). In the current study, we aimed to devise a novel mPCR assay for accurate detection and identification of pathogenic *Listeria*. The pan-genome analysis was performed to screen the genus- and species-specific genes. Using the identified genes, a mPCR method was developed to simultaneously detect *L. monocytogenes*, *L. ivanovii*, and nonpathogenic *Listeria*. Then the assay was used to evaluate the prevalence of pathogenic *Listeria* (including *L. monocytogenes* and *L. ivanovii*) in samples from *F. velutipes* plants.

## Materials and Methods

### Screening of Genus- and Species-Specific Novel Target Genes for *Listeria*

A total of 205 genome sequences of pathogenic bacteria were downloaded from the NCBI Genome Database (last accessed on 27 May 2019), including 165 *Listeria* sequences and 40 other common foodborne pathogens sequences. The specific information for the sequences is provided in Supplementary Table S1. The Pan-genome analysis was used to identify *Listeria* genus- and species-specific genes. Briefly, all nucleic acid sequences were annotated using Prokka v1.11 (Seemann, 2014). The output of Prokka was then used to construct a pan-genome analysis using Roary v3.11.2 (Page et al., 2015). A core genome was determined for each strain using a 99% cutoff, with a BLASTP identity cutoff of 85% (Chen et al., 2019; Pang et al., 2019). The absence/existence profile of all genes across strains was converted into a 0/1 matrix with local script. The *Listeria* genus-specific (or species-specific) genes were screened according to the following criteria: 100% presence in *Listeria* (or *L. monocytogenes*, or *L. ivanovii*) strains and no presence in non-*Listeria* (or non-target *Listeria* species) strains. Then these candidate targets were used as further screened against the nucleotide collection (nr/nt) databases using the online BLAST program^2^ and PCR verification to ensure its specificity. At the same time, the specific targets reported in the previous studies [including *prs* specific for *Listeria* genus (Doumith et al., 2004), *actA* (Gouin et al., 1995; Zhou and Jiao, 2005), *hly* (Paziak-Domańska et al., 1999), and *inlA* (Jung et al., 2003) specific for *L. monocytogenes*, and *iactA* specific for *L. ivanovii* (Gouin et al., 1995)] were also analyzed in the 0/1 matrix to evaluate their absence/existence profile.

### Bacterial Strains and Genomic DNA Extraction

A total of 126 bacteria strains were used in the study, which were obtained respectively from the Chinese Center for Disease Control and Prevention, the College of Food Science and Technology of Nanjing Agricultural University, the Guangdong Huankai Co., Ltd., the State Key Laboratory of Food Science and Technology of Nanchang University, and our laboratory (Table 1 and Supplementary Table S2). For DNA extraction, all strains were inoculated in brain heart infusion (BHI) medium (Guangdong Huankai Co., Ltd., Guangzhou, China) and incubated for 8–12 h at 37 °C. DNA was extracted using DNeasy Blood and Tissue kit (Qiagen, Shanghai, China), according to the manufacturer’s instructions. Extracted DNA was stored at –20 °C for PCR analysis.

**TABLE 1:** *Listeria* spp. and other common foodborne pathogenic strains used in this study to verify the specificity of candidate targets.

**Bacterial species**	**Serotype**	**Strain**	**Number of strains**	Source*	**PCR primer paris**				
					**SPP1**	**LM1**	**LIV1**	**LIV2**	**LIV3**
*L. monocytogenes*	1/2a		21	a	+	+	−	−	−
			8	a		+			
	3a	^1^ATCC 51782	1	b	+	+			
	3a		2	b	+	+			
	1/2c		15	a	+	+	−	−	−
	4b	ATCC 19115	1	d	+	+	−	−	−
	4b	^2^CMCC 54007	1	d	+	+			
	4b		15	a	+	+			
	4d	ATCC 19117	1	c	+	+			
	4e	ATCC 19118	1	c	+	+			
	4ab	Murray B	1	c	+	+			
	1/2b		20	a	+	+	−	−	−
	3b		1	c	+	+			
	7	^3^SLCC 2428	1	c	+	+			
	4a	ATCC 19114	1	c	+	+			
	4c		12	a	+	+	−	−	−
*L. innocua*	6a	ATCC 33090	1	d	+	−	−	−	−
*L. innocua*			3	a	+	−			
*L. ivanovii*	5	ATCC 19119	1	e	+	−	+	+	+
*L. seeligeri*	1/2b	^4^CICC 21671	1	c	+	−	−	−	−
*L. welshimeri*	6b	ATCC 35897	1	e	+	−	−	−	−
*L. grayi*		ATCC 19120	1	d	+	−	−	−	−
*Cronobacter sakazakii*		ATCC 29544	1	d	−	−	−	−	−
*Cronobacter sakazakii*			1	a	−	−	−	−	−
*Staphylococcus aureus*		ATCC 25923	1	d	−	−	−	−	−
*Staphylococcus aureus*		ATCC 29213	1	d	−	−	−	−	−
*Pseudomonas aeruginosa*		ATCC 9027	1	d	−	−	−	−	−
*Pseudomonas aeruginosa*		ATCC 27853	1	d	−	−	−	−	−
*Salmonella* Enteritidis		CMCC 50335	1	d	−	−	−	−	−
*Salmonella Typhimurium*		ATCC 14028	1	d	−	−	−	−	−
*Campylobacter jejuni*		ATCC 33291	1	d	−	−	−	−	−
*Escherichia coli*		ATCC 25922	1	d	−	−	−	−	−
*Shigella sonnei*		CMCC(B) 51592	1	d	−	−	−	−	−
*Bacillus cereus*		ATCC 14579	1	a	−	−	−	−	−
*Yersinia enterocolitica*			2	d	−	−	−	−	−
*Vibrio parahaemolyticus*		ATCC 33847	1	d	−	−	−	−	−
*Vibrio parahaemolyticus*		ATCC 17802	1	d	−	−	−	−	−

*a, our laboratory; b, Chinese Center for Disease Control and Prevention, China; c, College of Food Science and Technology, Nanjing Agricultural University, China; d, Guangdong Huankai Co., Ltd., China; e, State Key Laboratory of Food Science and Technology, Nanchang University, China.

*^1^ATCC, American Type Culture Collection, United States.*

*^2^CMCC, China Medical Culture Collection, China.*

*^3^SLCC, Seeliger’s Special *Listeria* Culture Collection, Germany.*

*^4^CICC, Center of Industrial Culture Collection, China. Result (±) indicate positive and negative signals.*

### Design and Validation of Genus- and Species-Specific Primers for *Listeria*

Primers for the candidate *Listeria* genus- and species-specific target genes were designed using the Oligo 7.0 software. Candidate primer sets were synthesized by Generay Biotech Co., Ltd., Shanghai, China. Primer specificity was tested by PCR analysis of strains from the laboratory collection (Table 1). Detection limits were determined using serially (10-fold) diluted cell suspensions of fresh cultures of *L. monocytogenes* ATCC 19115 and *L. ivanovii* ATCC 19119. Viable cells counts were determined by plating of 100 μL of 10^–6^, 10^–7^, and 10^–8^ dilutions of bacterial cultures on trypticase soy-yeast extract agar plates (Guangdong Huankai Co., Ltd., Guangzhou, China) and incubating overnight at 37 °C. PCR was performed using 2 μL of genomic DNA extracted from 1 mL different dilutions of cell mixtures as a template, with genus or species-specific primers. An equal volume of sterile distilled water was used instead of the template as a negative control. PCR thermal cycling involved an initial denaturation step at 95 °C for 10 min; followed by 35 cycles at 95 °C for 30 s, 55 °C for 30 s, and 72 °C for 30 s; and a final extension step at 72 °C for 10 min. PCR products were evaluated by 1.5% agarose electrophoresis.

### Multiplex PCR Conditions for Detection of Pathogenic *Listeria*

In the genus *Listeria*, only two species, *L. monocytogenes* and *L. ivanovii*, are human and animal pathogens. Therefore, a mPCR assay was devised by combining three specific primer sets targeting genes specific for the *Listeria* genus, *L. monocytogenes*, and *L. ivanovii* (Table 2). Multiplex PCR was performed in a volume of 25 μL; the reaction mixture contained 12.5 μL DreamTaq Green PCR Master Mix (2×), 0.1 mM dNTPs, 6 mM MgCl_2_, 1.5 U TaKaRa Ex Taq, 1.6 μM for *Listeria* genus-specific primers targeting *LMOSLCC2755_0944*, 0.32 μM for *L. monocytogenes*-specific primers targeting *LMOSLCC2755_0090* and 0.32 μM for *L. ivanovii*-specific primers targeting *queT_1*, and 4 μL of template. The genomic DNA of *L. monocytogenes* and *L. ivanovii* was added as a template for the positive control, and an equal volume of sterile distilled water was used instead of the template as a negative control. Multiplex PCR thermal cycling involved an initial denaturation step at 95 °C for 10 min; followed by 35 cycles at 95 °C for 30 s, 58.1°C for 30 s, and 72 °C for 30 s; and a final extension step at 72 °C for 10 min. The mPCR products were analyzed after electrophoresis on 2.5% agarose gel under ultraviolet light illumination.

**TABLE 2:** Specific genes and primers for PCR identification of *Listeria* spp. and pathogenic *Listeria*.

**Species**	**Name of target genes**	**Encoded protein**	**Primer set name**	**Sequences (5′–3′)**	**Product size (bp)**
*Listeria* spp.	*LMOSLCC2755_0944*	Non-heme iron-containing ferritin	SPP1	CAGTAGACACAAAGGAAT	427
				GCTTTGAACATCCAGATA	
*L. monocytogenes*	*LMOSLCC2755_0090*	Transcriptional regulator belonging to the MerR family	LM1	GCTTAATAACCCCTGACCG	260
				AATCCCAATCTTCCTAACCAC	
*L. ivanovii*	*NCTC12701_01099*	Hypothetical protein	LIV1	GCTAAAAACGACATAGAGG	264
				CTCTTCTAACACAATCACT	
	*queT_1*	Queuosine precursor ECF transporter S component QueT	LIV2	AGCCATCCAACTACGAAT	144
				GACCCACCGCCTATAAAA	
	*gmuC_3*	PTS system oligo-beta-mannoside-specific EIIC component	LIV3	TATTTGGACCACCGCATTA	452
				TAGCAGGAACATCTTCACTT	

### Evaluation of Specificity and Detection Limit of mPCR Assay

The specificity of mPCR with novel specific primers was verified using 33 bacteria strains (including 17 *Listeri*a strains and 16 non-*Listeria* strains) (Supplementary Table S2). Primer sets that amplified bands of the expected length from the corresponding strains of *Listeria* but not from non-*Listeria* strains were considered species-specific. For the detection limit evaluation, *Listeria* cultured overnight were diluted 10-fold in normal saline and mixed. Purified genomic DNA extracted from different dilutions of cell suspensions was used as the mPCR template (4 μL). An equal volume of sterile distilled water was included in the mPCR mixture instead of the template, as a negative control. The sensitivity of the PCR was determined by electrophoresis, as described in section “Multiplex PCR Conditions for Detection of Pathogenic *Listeria*.”

### Artificial Contamination Experiments

Artificial contamination experiments were performed as previously described (Chen et al., 2019). Briefly, *L. monocytogenes* ATCC 19115 and *L. ivanovii* ATCC 19119 were cultured overnight, and the cell concentration was determined by plating. *F. velutipes* samples (10 g) were mixed by 88 mL of the BHI medium, and then 2 mL *Listeria* mixtures were added with the final inoculum concentration ranging from 7.6 × 10^0^ to 7.6 × 10^4^ CFU/10 g. Next, the mixtures were incubated at 37 °C for 4 h, 6 h, 8 h, 10 h, or 12 h. Genomic DNA was extracted at the indicated time points from 1 mL of sample, and then analyzed by mPCR.

### Interference Evaluation

To validate the accuracy and scope for interference in the mPCR assay, *L. monocytogenes* ATCC 19115, *L. ivanovii* ATCC19119, and three common pathogens (*Salmonella* Enteritidis CMCC 50335, *Staphylococcus aureus* ATCC 25923, and *Escherichia coli* ATCC 25922) were used. The strains were cultured in the BHI broth overnight and serially diluted (10-fold) with normal saline. The density of *Listeria* cells was adjusted to 10^6^ CFU/mL. *Listeria* cultures were individually mixed with the interference testing strain in ratios of 1:10^2^, 1:10, 1:1, 10:1, and 10^2^:1. Genomic DNA was extracted from the mixtures. Meanwhile, the genomic DNA from *Listeria* cultures without interference strains was used as a template for the positive control. The scope of mPCR assay to overcome interference was evaluated by 2.5% agarose gel electrophoresis.

### Detection of Pathogenic *Listeria* in *F. velutipes* Plants

To validate the detection ability of mPCR, a total of 129 samples were collected from *F. velutipes* plants in Guangdong Province (China). The sampled sites included composting sites (compost and sterile compost, *n* = 15), mycelium culture rooms (cultures and shelf surfaces, *n* = 18), mycelium stimulation rooms (mycelium stimulation machinery and floor, *n* = 18), fruiting body cultivation rooms (drains, shelf surfaces, and *F. velutipes*, *n* = 27), and harvesting rooms (packaging machinery surfaces, scales, conveyor belts, cutler surfaces, packaged *F. velutipes*, floor, drains, and workers, *n* = 51). The sites at the *F. velutipes* plants were randomly sampled, and the samples were transported on ice to the laboratory for immediate analysis. The conventional culture method was used to test for the presence of *Listeria* as previously described (Chen et al., 2019). Briefly, 25 g of each sample was randomly weighed-out, added to 225 mL of *Listeria* enrichment broth 1 (LB1, Guangdong Huankai Co., Ltd., Guangzhou, China), and incubated at 30 °C for 24 h. After incubation, 100 μL of LB1 enrichment culture was transferred to 10 mL of *Listeria* enrichment broth 2 (LB2, Guangdong Huankai Co., Ltd., Guangzhou, China), and incubated at 30 °C for 24 h. A loopful (approximately 10 μL) of the LB1 enrichment culture was streaked onto *Listeria* selective agar plates (Guangdong Huankai Co., Ltd., Guangzhou, China), and incubated at 37 °C for 48 h. At least three presumptive colonies were selected for the identification of *Listeria* using the Microgen ID *Listeria* identification system (Microgen, Camberley, United Kingdom), according to the manufacturer’s instructions. Meanwhile, 1 mL of LB1 enrichment culture was collected from each sample at 12 h. Genomic DNA was extracted from LB1 enrichment cultures for mPCR.

### Data Analysis

The Kappa coefficient (κ) was used to evaluate the performance of the developed mPCR assay. Since the true pathogenic *Listeria* status in naturally contamination samples was unknown, this calculation method assumed that conventional culture analysis was the true value. The calculation method was performed as previously described (Tao et al., 2017).

## Results

### Novel Target Genes Specific for the *Listeria* Genus and Species

In the current study, 205 genomic sequences (Supplementary Table S1) were included in pan-genome analysis to identify novel molecular targets for the detection and differentiation of *L. monocytogenes* and *L. ivanovii*, and nonpathogenic *Listeria* species. After filtering using pan-genome and PCR analysis, five novel *Listeria*-specific targets, including *LMOSLCC2755_0944* (165/165) specific for *Listeria* genus, *LMOSLCC2755_0090* (128/128) specific for *L. monocytogenes*, *NCTC12701_01099* (9/9), *queT_1* (9/9), and *gmuC_3* (9/9) specific for *L. ivanovii*, were uniquely present in all target strains, but not in non-target strains (Tables 1, 3 and Supplementary Figure S1). The *Listeria*-specific targets reported in the previous studies, including *prs*, *actA*, *hly*, *inlA*, and *iactA*, were used to specifically target strains and detected these targets in the current study, with 98.8% (163/165), 97.7% (125/128), 99.2% (127/128), 71.9% (92/128), and 55.6% (5/9) of target strains harboring the marker, respectively (Table 3).

**TABLE 3:** Presence profile of novel *Listeria*-specific targets for target and non-target strains.

**Species**	**Related gene**	**Presence profile**		**Source**
		**In target**	**In non-target**	
*Listeria* spp.	*LMOSLCC2755_0944*	165 (100%)	0 (0)	Our study
	*prs*	163 (98.8%)	0 (0)	Doumith et al., 2004
*L. monocytogenes*	*LMOSLCC2755_0090*	128 (100%)	0 (0)	Our study
	*actA*	125 (97.7%)	0 (0)	Gouin et al., 1995; Zhou and Jiao, 2005
	*hly*	127 (99.2%)	0 (0)	Paziak-Domańska et al., 1999
	*inlA*	92 (71.9%)	0 (0)	Jung et al., 2003
*L. ivanovii*	*NCTC12701_01099*	9 (100%)	0 (0)	Our study
	*queT_1*	9 (100%)	0 (0)	Our study
	*gmuC_3*	9 (100%)	0 (0)	Our study
	*iactA*	5 (55.6%)	0 (0)	Gouin et al., 1995

As shown in Table 2, for the *Listeria* genus, only one gene (*LMOSLCC2755_0944*, encoding a non-heme iron-containing ferritin) was identified as genus-specific. The four other genes were species-specific: one gene (*LMOSLCC2755_0090*, encoding a transcriptional regulator from the MerR family) was specific for *L. monocytogenes*, and three genes (*NCTC12701_01099*, encoding a hypothetical protein; *queT_1*, encoding a queuosine precursor ECF transporter S component QueT; and *gmuC_3*, encoding a PTS system oligo-beta-mannoside-specific EIIC component) were specific for *L. ivanovii*.

### Selection of Novel Species-Specific Genes for mPCR

**FIGURE 1:**
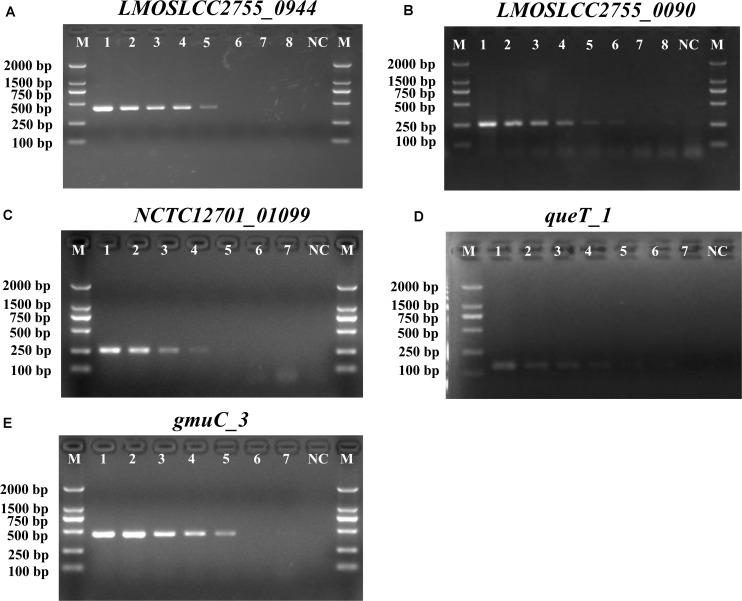
PCR detection sensitivity of novel targets specific for *Listeria*. **(A)** *LMOSLCC2755_0944* specific for the *Listeria* spp.; **(B)** *LMOSLCC2755_0090* specific for *L. monocytogenes*; **(C)** *NCTC12701_01099*; **(D)** *queT_1*; and **(E)** *gmuC_3* specific for *L. ivanovii*. Lane M: DL2000 DNA standard marker; **(A)** lanes 1–8: the bacterial culture concentration per PCR assay from 10^7^ to 10^0^ CFU/mL; **(B)** lanes 1–8: the bacterial culture concentration per PCR assay from 10^8^ to 10^1^ CFU/mL; **(C–E)** lanes 1–7: the bacterial culture concentration per PCR assay from 10^7^ to 10^1^ CFU/mL; lane NC: negative control.

Before devising a mPCR assay, the sensitivity of primers for each target gene was evaluated in an individual PCR assay. The results are shown in Supplementary Table S3 and Figure 1. The detection limit of these primer pairs was 10^3^–10^4^ CFU/mL. Considering the amplicon lengths for different genes, the sensitivity of species-specific primers, and the competition and inhibition of primer sets in the reaction system, a combination of suitable primer pairs (SPP1, LM1, and LIV2) targeting the *LMOSLCC2755_0944*, *LMOSLCC2755_0090*, and *queT_1* genes (respectively) was selected for the mPCR assay.

### Evaluation of the Specificity and Sensitivity of the mPCR Assay for *Listeria* spp.

The results of the specificity of mPCR assay with novel specific primers are shown in Supplementary Table S2. The genus-specific target band of 427 bp was only obtained with *Listeria* spp. as the template. Further, species-specific bands were also obtained for the *L. monocytogenes* and *L. ivanovii* strains tested. No product bands were obtained with the 16 non-*Listeria* strains tested, and no cross-reactivity of the mPCR was observed.

**FIGURE 2:**
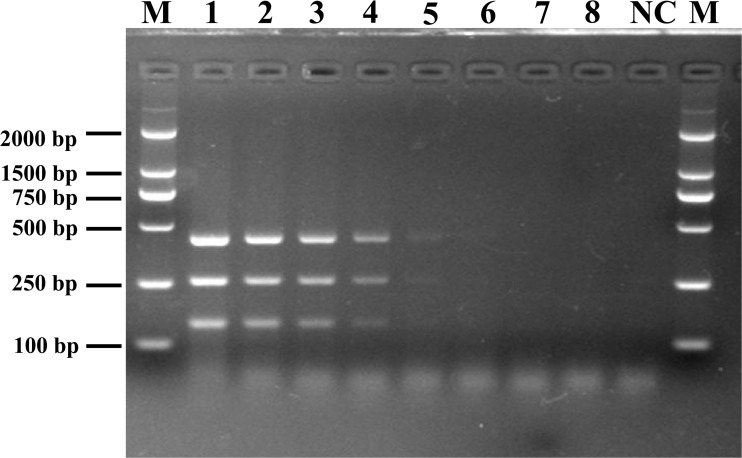
Detection limit of mPCR for pure culture from pathogenic *Listeria.* Lane M: DL2000 DNA standard marker; lanes 1–8: the bacterial culture concentration per PCR assay from 5.4 × 10^7^ to 5.4 × 10^0^ CFU/mL; lane NC: negative control.

To determine the detection limit of the novel assay, DNA was extracted from different dilutions of *Listeria* spp. cultures and used as the reaction template. Following mPCR detection, three specific fragments of 427 bp (SPP1), 260 bp (LM1), and 144 bp (LIV2) were obtained for cell concentrations of 10^7^ to 10^3^ CFU/mL (Figure 2). The analysis suggested that the detection limits of the mPCR assay were 2.0 × 10^3^ CFU/mL for *L. monocytogenes* and 3.4 × 10^3^ CFU/mL for *L. ivanovii*.

### Multiplex PCR Detection of *Listeria* in Artificially Contaminated *F. velutipes* Samples

*F. velutipes* samples were spiked with *Listeria* cells at the inoculum levels of 7.6 × 10^4^, 7.6 × 10^3^, 7.6 × 10^2^, 7.6 × 10^1^, and 7.6 × 10^0^ CFU/10 g, and the enrichment cultures were followed for up to 12 h. As shown in Supplementary Table S4, the detection outcomes of mPCR varied depending on the enrichment time. The detection limit of mPCR after 4-h, 6-h, 8-h, 10-h, and 12-h enrichment was 7.6 × 10^4^, 7.6 × 10^3^, 7.6 × 10^2^, 7.6 × 10^1^, and 7.6 × 10^0^ CFU/10 g *F. velutipes*, respectively (Supplementary Table S4).

### Interference Testing of the mPCR Assay

**FIGURE 3:**
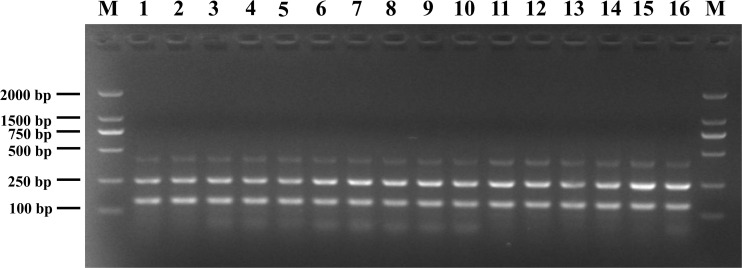
Anti-interference tests of mPCR detection of pathogenic *Listeria* with *S.* Enteritidis CMCC 50335 (lanes 1–5), *S. aureus* ATCC 25923 (lanes 6–10), and *E. coli* ATCC 25922 (lanes 11–15) at different mixing proportion. Lane M: DL2000 DNA standard marker; lanes 1–5, 6–10, 11–15: the ratio of target bacteria to interfering bacteria at 1:10^2^, 1:10, 1:1, 10:1, and 10^2^:1; lane 16: positive control.

The susceptibility of the mPCR assay to interference by non-target DNA was determined by mixing pathogenic *Listeria* and non-*Listeria* strains in different ratios (Figure 3). Three clear bands were generated for mixtures of all strains tested, and the brightness of these bands was comparable with that obtained from analyzing pure pathogenic *Listeria* cultures. This indicated that the presence of *S.* Enteritidis CMCC 50335, *S. aureus* ATCC 25923, and *E. coli* ATCC 25922 did not interfere with the detection of pathogenic *Listeria*.

### Application of the mPCR Assay for the Analysis of *F. velutipes* Plants Samples

To verify the practicality and effectiveness of the developed mPCR method, we next used the assay to detect *Listeria* in 129 non-spiked *F. velutipes* plants samples (Supplementary Table S5). In the analyzed samples, the prevalence of *Listeria* spp. and *L. monocytogenes* was 58.1% and 41.1%, respectively. The contaminated sites included the composting area, mycelium scraping equipment surfaces, floor, fruiting body cultivation room, and harvesting room. No pathogenic *Listeria* spp. were detected at the composting phase and mycelium culture room samples. The bacteria appeared to originate from the mycelium scraping machinery, which contaminated the product and downstream packaging equipment.

**FIGURE 4:**
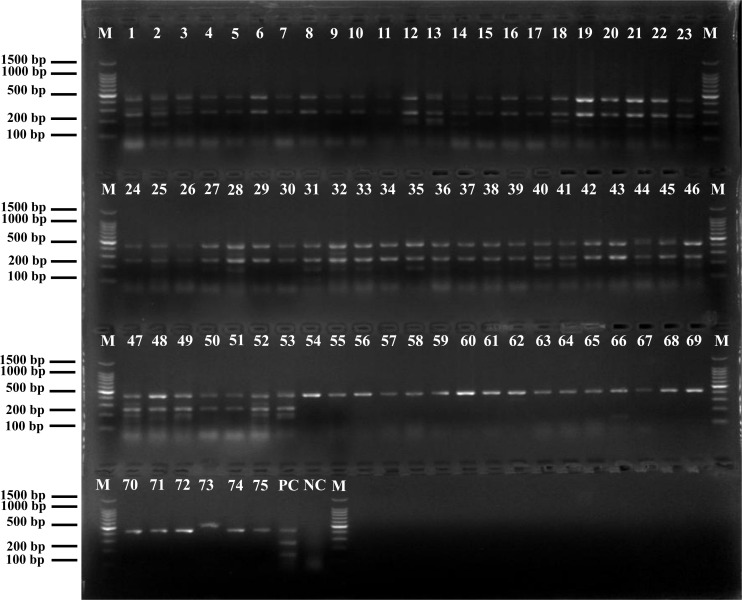
Positive results of mPCR in natural *Flammulina velutipes* plants samples. Lane M: DL2000 DNA standard marker; lanes 1–53: positive samples containing *L. monocytogenes*; lanes 54–75: positive samples containing non-pathogenic *Listeria* spp.; lane PC: positive control; lane NC: negative control.

The results of positive identification of *Listeria* spp. by traditional methods in 75 samples also analyzed by mPCR are summarized in Figure 4. Among 174 strains identified as *Listeria* spp., 79 strains (45.4%) were identified as *L. monocytogenes* and 95 strains (54.6%) were identified as nonpathogenic *Listeria* (including 91 *L. innocua* and 4 *L. grayi*). In 129 *F. velutipes* plant samples, no *L. ivanovii, L. seeligeri*, and *L. welshimeri* were detected. A comparison of the mPCR outcomes with the results of conventional culture method is shown in Table 4. For detection of *Listeria* spp., the sensitivity, specificity, and efficacy of mPCR were 98.7%, 98.1%, and 98.5%, respectively. For detection of *L. monocytogenes*, the sensitivity, specificity, and efficacy of mPCR were 98.2%, 98.7%, and 98.5%, respectively. Only one false-positive and one false-negative result were obtained for 129 samples tested. The calculated κ factors for *Listeria* spp., *L. monocytogenes*, and *L. ivanovii* were 0.97, 0.97, and 1, respectively, indicating that the mPCR outcomes were highly consistent with those of the conventional culture method.

**TABLE 4:** Statistical evaluation of mPCR assay for natural *Flammulina velutipes* plants samples comparison with conventional culture method.

**Species**	**Samples number**	**Multiplex PCR results**	**Conventional culture method results**		**Sensitivity (%)**	**Specificity (%)**	**Efficiency (%)**	**Predictive value (%)**		**κ**
			**+**	**−**				**+**	**−**	
*Listeria* spp.	129	+	75 (a)	1 (b)	98.7	98.1	98.5	98.7	98.1	0.97
		−	1 (c)	52 (d)						
*L. monocytogenes*		+	53 (a)	1 (b)	98.2	98.7	98.5	98.2	98.7	0.97
		−	1 (c)	74 (d)						
*L. ivanovii*		+	0 (a)	0 (b)	100	100	100	0	100	1
		−	0 (c)	129 (d)						

*^*a*^True positive; ^*b*^false positive; ^*c*^false negative; ^*d*^true negative.*

## Discussion

In the current study, we devised a novel mPCR method for detection of pathogenic *Listeria* spp. in food. The method focuses on new genus- and species-specific gene targets, and is highly specific and sensitive. It may inform the development of highly sensitive PCR approaches for the detection of *Listeria* in food samples and food processing plants.

*Listeria* spp. is widely found in food, especially in edible mushrooms, with the detection rate as high as 21.2% (Chen et al., 2018). Therefore, the development of PCR-based detection methods for species-specific classification would provide an independent means for confirming species identity (Ryu et al., 2013; Liu et al., 2015). The current PCR detection methods for *Listeria* species target virulence genes, or 16S rRNA and 23S rRNA genes. However, the lack of genes or mutations in *Listeria* strains can result in no identification or misidentification, posing a potential threat of food poisoning (Nishibori et al., 1995). As numerous complete microbial genome sequences become available with the development of sequencing technologies and bioinformatics, many researchers are committed to the search for novel specific gene targets, to replace current target genes that exhibit poor specificity (Tao et al., 2017; Sheng et al., 2018). Previously, specific target genes for *Listeria* were identified by DNA hybridization or using the BLAST program (Michel et al., 2004; Tao et al., 2017). Identification of a specific target by using DNA arrays involves the synthesis of probes, PCR amplification, array construction, and hybridization (Michel et al., 2004). The BLAST search method of specific target screening usually involves nucleotide sequence similarity comparisons with one or only few representative genomic sequences (Tao et al., 2017). With the rapid development of bioinformatics in recent years, using the pan-genome analysis to identify specific targets is more economical, convenient, and effective than other molecular target screening methods. In the current study, we identified gene targets specific for the *Listeria* genus and species via pan-genome analysis using a large number of genome sequences (*n* = 205). Through pan-genome and PCR analysis, five novel *Listeria*-specific targets were 100% specific for the targeted *Listeria* genomes and did not detect non-target *Listeria* and non-*Listeria* genomes. However, The *Listeria*-specific targets reported in the previous studies, including *prs*, *actA*, *hly*, *inlA*, and *iactA*, were present in 98.8%, 97.7%, 99.2%, 71.9%, and 55.6% of the target strains, respectively (Table 3). In addition, the detection limits of the corresponding primer pairs (10^3^–10^4^ CFU/mL) of these novel target genes were comparable with those of existing molecular detection targets (Chiang et al., 2012; Tao et al., 2017). Therefore, these *Listeria*-specific targets obtained by this approach have better specificity, and their sensitivity can meet the needs of existing molecular detection methods, so these novel targets can represent unique detection targets for monitoring of pathogenic *Listeria* in food and its production environment.

The presence of pathogenic *Listeria* strains in *F. velutipes* is an emerging public health hazard (Chen et al., 2018). Therefore, rapid detection of pathogenic *Listeria* is crucial for implementing optimal sterilization procedures for decontamination during the preparation of *F. velutipes* products. The mPCR assay developed in the current study combines three specific primer sets (SPP1, LM1, and LIV2) based on novel molecular markers (*LMOSLCC2755_0944*, *LMOSLCC2755_0090*, and *queT_1*, respectively) and allows simultaneous identification of pathogenic *Listeria* species. To increase the accuracy of *Listeria* identification, the *Listeria* genus-specific SPP1 primer set was also included. This enhances the specificity of the mPCR, especially when high concentrations of non-*Listeria* bacteria are present in a sample. The minimum detection limits of the assay were 2.0 × 10^3^ CFU/mL for *L. monocytogenes* and 3.4 × 10^3^ CFU/mL for *L. ivanovii* when pure enriched cultures were analyzed, which is comparable to those of mPCRs reported in previous studies (Chiang et al., 2012; Thapa et al., 2013; Sheng et al., 2018). In addition, the developed mPCR method detected pathogenic *Listeria* in artificially contaminated *F. velutipes* samples in the range of 7.6 × 10^4^–7.6 × 10^0^ CFU/10 g after 4–12 h of enrichment culture. These observations indicated that the devised mPCR assay could be used to detect pathogenic *Listeria* in samples more rapidly (The overall assay time, including 4–12 h pre-enrichment, DNA extraction and PCR assay, only took 5–17 h) than by using the standard culture method (4–7 days).

We also used the novel mPCR assay to analyze 129 samples from *F. velutipes* plants. The assay was highly sensitive, specific, and efficient. High *κ* values of the assay (0.97 for *Listeria* spp., 0.97 for *L. monocytogenes*, and 1 for *L. ivanovii*) indicated good correspondence between the assay and a conventional culture method. In an earlier preliminary study, we found that the prevalence of *Listeria* spp. and *L. monocytogenes* in *F. velutipes* production plants is 52.5% and 18.6%, respectively (Chen et al., 2014). In the current study, we noted a relatively high incidence of *Listeria* contamination (58.1% for *Listeria* spp. and 41.1% for *L. monocytogenes*). A possible reason for such high incidence is that many samples were collected on the floor and drains in this study, and the cross-contamination of *L. monocytogenes* easily occurred in these collection sites during the production process of *F. velutipes*. Among 174 strains identified as *Listeria* spp., approximately 54.6% strains were nonpathogenic *Listeria* species (including 91 *L. innocua* and 4 *L. grayi*). *L. innocua* occurred more frequently than *L. monocytogenes* in the plant and related samples, which was consistent with previous findings (Chen et al., 2014). The presence of *L. innocua* may hamper the detectability of *L. monocytogenes*, by inhibiting the pathogen via excessive growth and production of inhibitory compounds (Cornu et al., 2002; Zitz et al., 2011). Therefore, the presence of nonpathogenic *Listeria*, especially *L. innocua*, may accompany *L. monocytogenes* contamination (Pagadala et al., 2012). Consequently, a positive band for the *Listeria* genus-specific target gene in the novel mPCR assay may be an indicator of the presence of *L. monocytogenes.* Finally, isolation of pathogenic *Listeria* strains from *F. velutipes* plants indicated a potential risk of contracting listeriosis when consuming this mushroom. In most cases, *L. monocytogenes* is inactivated by cooking and heating. Therefore, edible mushrooms should be fully heated before use and cross-contamination between foods should be avoided.

## Conclusion

In conclusion, we used pan-genome analysis to identify five novel genus- and species-specific targets for *Listeria* detection. We then devised a mPCR assay based on these new targets for simultaneous detection of *L. monocytogenes*, *L. ivanovii*, and other *Listeria* spp. with high specificity and sensitivity. The results of the assay were consistent with the results of traditional culture methods. Hence, the developed assay can be applied for rapid screening and detection of pathogenic *Listeria* in foods and food processing environments, providing accurate results to inform effective monitoring measures to improve the microbiological safety of foods.

## Data Availability Statement

The original contributions presented in the study are included in the article/Supplementary Material, further inquiries can be directed to the corresponding author/s.

## Author Contributions

QW, JZ, QY, and MC conceived and designed the experiments. FL, LX, and YZ performed the experiments. FL, JW, SW, and HZ analyzed the data. YD, QG, and XW contributed reagents, materials, and analysis tools. FL contributed to the writing of the manuscript. All authors contributed to the article and approved the submitted version.

## Conflict of Interest

The authors declare that the research was conducted in the absence of any commercial or financial relationships that could be construed as a potential conflict of interest.

## Footnotes

1 https://www.ncbi.nlm.nih.gov/genome/

2 https://blast.ncbi.nlm.nih.gov/Blast.cgi
