# Supplementary material for: Multiplex PCR for the Identification of Pathogenic Listeria in Flammulina velutipes Plant Based on Novel Specific Targets Revealed by Pan-Genome Analysis
Source: Front Microbiol. 2021 Jan 15;11:634255. doi: 10.3389/fmicb.2020.634255 (PMC7843925; doi:10.3389/fmicb.2020.634255)
Supplement: Supplementary file 1 [file Data_Sheet_1.doc]

**Supplementary Material (1)**

**Multiplex PCR for the Identification of Pathogenic *Listeria* in** ***Flammulina velutipes* Plant** **Based on Novel Specific Targets Revealed by Pan-genome Analysis**

Fan Li ^1, 2 #^, Qinghua Ye ^1 #^, Moutong Chen ^1, *^, Jumei Zhang ^1^, Liang Xue ^1^, Juan Wang ^3^, Shi Wu ^1^, Haiyan Zeng ^1^, Qihui Gu ^1^, Youxiong Zhang ^1^, Xianhu Wei ^1^, Yu Ding ^4 *^, Qingping Wu ^1 *^

^1^ Guangdong Provincial Key Laboratory of Microbial Safety and Health, State Key Laboratory of Applied Microbiology Southern China, Guangdong Institute of Microbiology, Guangdong Academy of Sciences, Guangzhou, China

^2^ School of Biology and Biological Engineering, South China University of Technology, Guangzhou, China

^3^ College of Food Science, South China Agricultural University, Guangzhou, China

^4^ Department of Food Science and Technology, Jinan University, Guangzhou, China

**^*^ Corresponding author:**

Qingping Wu

1. mail: [wuqp203@163.com](mailto:wuqp203@163.com)

Tel: +86-20-87688132; fax: +86-20-87688132

Address: Guangdong Institute of Microbiology, Yard 100#, Xianlie Zhong Lu, Yuexiu District, 510070 Guangzhou, P.R.China

Yu Ding

E-mail: dingyu@jnu.edu.cn

Tel: +86-20-85222379

Address: Department of Food Science & Technology, Institute of Food Safety and Nutrition, Jinan University, Huangpu Ave. 601, Guangzhou 510632, China

^#^ Fan Li, Qinghua Ye, and Moutong Chen contribute to the manuscript equally.

**Table S1.** Information for *Listeria* and non-*Listeria* isolates analyzed during this study.

| Organism | Strain | Assembly | Size (Mb) | GC% | Scaffolds |
| --- | --- | --- | --- | --- | --- |
| *Listeria monocytogenes* | F2365 | GCA_000008285.1 | 2.90519 | 38 | 1 |
| *Listeria monocytogenes* | HCC23 | GCA_000021185.1 | 2.97621 | 38.2 | 1 |
| *Listeria monocytogenes* | J1-220 | GCA_000195395.5 | 3.03227 | 37.9 | 1 |
| *Listeria monocytogenes* | J1816 | GCA_000195435.4 | 2.94746 | 38 | 1 |
| *Listeria monocytogenes* | SLCC2755 | GCA_000197755.2 | 3.02393 | 38.06 | 2 |
| *Listeria monocytogenes* | L99 | GCA_000209755.1 | 2.9792 | 38.2 | 1 |
| *Listeria monocytogenes* | [SLCC2482](https://www.ncbi.nlm.nih.gov/genome/159?genome_assembly_id=159703) | GCA_000210795.2 | 2.98679 | 37.96 | 2 |
| *Listeria monocytogenes* | SLCC2372 | GCA_000210815.2 | 3.02291 | 37.96 | 2 |
| *Listeria monocytogenes* | Scott A | GCA_000212455.1 | 3.02182 | 37.9 | 1 |
| *Listeria monocytogenes* | M7 | GCA_000218305.1 | 2.97616 | 38.2 | 1 |
| *Listeria monocytogenes* | SLCC2540 | GCA_000306905.1 | 2.97696 | 37.9 | 1 |
| *Listeria monocytogenes* | SLCC7179 | GCA_000306985.1 | 2.88223 | 38 | 1 |
| *Listeria monocytogenes* | SLCC2479 | GCA_000307005.1 | 2.97217 | 38 | 1 |
| *Listeria monocytogenes* | ATCC 19117 | GCA_000307025.1 | 2.9518 | 38 | 1 |
| *Listeria monocytogenes* | SLCC5850 | GCA_000307045.1 | 2.90714 | 38 | 1 |
| *Listeria monocytogenes* | SLCC2376 | GCA_000307065.1 | 2.84018 | 38.3 | 1 |
| *Listeria monocytogenes* | L312 | GCA_000307085.1 | 2.91235 | 38.1 | 1 |
| *Listeria monocytogenes* | SLCC2378 | GCA_000307615.1 | 2.94136 | 38 | 1 |
| *Listeria monocytogenes* | [LL195](https://www.ncbi.nlm.nih.gov/genome/159?genome_assembly_id=159685) | GCA_000318055.1 | 2.90466 | 38 | 1 |
| *Listeria monocytogenes* | NCCP No. 15743 | GCA_000397145.1 | 2.86066 | 38.1 | 5 |
| *Listeria monocytogenes* | R2-502 | GCA_000438585.1 | 3.0916 | 37.86 | 2 |
| *Listeria monocytogenes* | C1-387 | GCA_000438605.1 | 2.98895 | 38 | 1 |
| *Listeria monocytogenes* | J2-064 | GCA_000438625.1 | 2.94322 | 38 | 1 |
| *Listeria monocytogenes* | J2-031 | GCA_000438645.1 | 2.95891 | 38 | 1 |
| *Listeria monocytogenes* | [J1-108](https://www.ncbi.nlm.nih.gov/genome/159?genome_assembly_id=299336) | GCA_000438665.1 | 2.98189 | 38 | 1 |
| *Listeria monocytogenes* | N1-011A | GCA_000438685.2 | 3.2433 | 37.95 | 2 |
| *Listeria monocytogenes* | J1776 | GCA_000438705.2 | 3.00952 | 37.94 | 2 |
| *Listeria monocytogenes* | [J1817](https://www.ncbi.nlm.nih.gov/genome/159?genome_assembly_id=299339) | GCA_000438725.2 | 3.00952 | 37.94 | 2 |
| *Listeria monocytogenes* | J1926 | GCA_000438745.2 | 3.00951 | 37.94 | 2 |
| *Listeria monocytogenes* | LS642 | GCA_000465735.1 | 2.94347 | 37.9 | 24 |
| *Listeria monocytogenes* | LS643 | GCA_000465755.1 | 2.88131 | 37.9 | 30 |
| *Listeria monocytogenes* | LS542 | GCA_000465815.1 | 2.93073 | 37.9 | 45 |
| *Listeria monocytogenes* | 08-6569 | GCA_000513595.1 | 3.03262 | 38 | 1 |
| *Listeria monocytogenes* | 81-0861 | GCA_000513615.1 | 2.9744 | 38 | 1 |
| *Listeria monocytogenes* | [08-6997](https://www.ncbi.nlm.nih.gov/genome/159?genome_assembly_id=279171) | GCA_000513635.1 | 3.03262 | 38 | 1 |
| *Listeria monocytogenes* | [10-0815](https://www.ncbi.nlm.nih.gov/genome/159?genome_assembly_id=279172) | GCA_000513655.1 | 3.03262 | 38 | 1 |
| *Listeria monocytogenes* | 10-1047 | GCA_000513675.1 | 2.99821 | 38 | 1 |
| *Listeria monocytogenes* | 88-0478 | GCA_000513695.1 | 2.99435 | 38 | 1 |
| *Listeria monocytogenes* | [WSLC1001](https://www.ncbi.nlm.nih.gov/genome/159?genome_assembly_id=159697) | GCA_000568475.1 | 2.95124 | 38 | 1 |
| *Listeria monocytogenes* | WSLC1042 | GCA_000568935.1 | 2.94217 | 38 | 1 |
| *Listeria monocytogenes* | Lm60 | GCA_000746625.1 | 2.98959 | 38 | 1 |
| *Listeria monocytogenes* | L2074 | GCA_001027065.1 | 2.89719 | 38 | 1 |
| *Listeria monocytogenes* | L2625 | GCA_001027125.1 | 2.89659 | 38.1 | 1 |
| *Listeria monocytogenes* | L2624 | GCA_001027165.1 | 2.9325 | 38 | 1 |
| *Listeria monocytogenes* | L2676 | GCA_001027205.1 | 2.94729 | 38 | 1 |
| *Listeria monocytogenes* | L2626 | GCA_001027245.1 | 2.90749 | 38 | 1 |
| *Listeria monocytogenes* | WSLC 1018 | GCA_001454845.1 | 2.94251 | 38 | 1 |
| *Listeria monocytogenes* | WSLC 1019 | GCA_001454865.1 | 2.83987 | 38.2 | 1 |
| *Listeria monocytogenes* | WSLC 1047 | GCA_001454925.1 | 2.95075 | 38 | 1 |
| *Listeria monocytogenes* | Lm 3163 | GCA_001483405.1 | 2.92775 | 38 | 1 |
| *Listeria monocytogenes* | Lm N1546 | GCA_001483445.1 | 3.03922 | 37.97 | 2 |
| *Listeria monocytogenes* | [Lm105](https://www.ncbi.nlm.nih.gov/genome/159?genome_assembly_id=281089) | GCA_001709365.1 | 2.99887 | 37.8 | 14 |
| *Listeria monocytogenes* | [Lm233](https://www.ncbi.nlm.nih.gov/genome/159?genome_assembly_id=281095) | GCA_001709465.1 | 2.93946 | 37.8 | 44 |
| *Listeria monocytogenes* | [Lm231](https://www.ncbi.nlm.nih.gov/genome/159?genome_assembly_id=281102) | GCA_001709625.1 | 2.96172 | 37.9 | 15 |
| *Listeria monocytogenes* | Lm218 | GCA_001709645.1 | 3.02317 | 37.9 | 29 |
| *Listeria monocytogenes* | A520 | GCA_001710165.1 | 2.9459 | 37.8 | 14 |
| *Listeria monocytogenes* | A147 | GCA_001710805.1 | 3.03608 | 37.8 | 285 |
| *Listeria monocytogenes* | Lm221 | GCA_001711245.1 | 2.94996 | 37.8 | 20 |
| *Listeria monocytogenes* | A507 | GCA_001711645.1 | 3.05302 | 37.8 | 36 |
| *Listeria monocytogenes* | NRRL B-33228 | GCA_001759965.1 | 2.98005 | 37.9 | 24 |
| *Listeria monocytogenes* | NRRL B-33402 | GCA_001760545.1 | 2.95671 | 37.8 | 16 |
| *Listeria monocytogenes* | NRRL B-33578 | GCA_001760765.1 | 3.0042 | 37.8 | 49 |
| *Listeria monocytogenes* | NRRL B-33804 | GCA_001761135.1 | 2.86036 | 37.9 | 13 |
| *Listeria monocytogenes* | NRRL B-33812 | GCA_001761245.1 | 3.03536 | 37.8 | 37 |
| *Listeria monocytogenes* | 2008-911 | GCA_001866445.1 | 3.1066 | 37.8 | 115 |
| *Listeria monocytogenes* | VIMVR081 | GCA_001889585.1 | 3.05445 | 38 | 1 |
| *Listeria monocytogenes* | VIMHA007 | GCA_001889645.1 | 2.9913 | 38 | 1 |
| *Listeria monocytogenes* | HPB913 | GCA_001913195.1 | 3.00535 | 38 | 1 |
| *Listeria monocytogenes* | LM41 | GCA_001984485.1 | 2.96311 | 37.8 | 11 |
| *Listeria monocytogenes* | 10-092876-0168 | GCA_001998945.1 | 3.07209 | 37.9 | 1 |
| *Listeria monocytogenes* | 10-092876-1063 LM3 | GCA_001998985.1 | 2.91395 | 38 | 1 |
| *Listeria monocytogenes* | 10-092876-0055 LM4 | GCA_001999005.1 | 2.98969 | 37.9 | 1 |
| *Listeria monocytogenes* | 10-092876-0731 LM5 | GCA_001999025.1 | 2.99679 | 38 | 1 |
| *Listeria monocytogenes* | 10-092876-1155 LM6 | GCA_001999045.1 | 2.97445 | 38 | 1 |
| *Listeria monocytogenes* | 10-092876-1547 LM7 | GCA_001999065.1 | 2.92201 | 38 | 1 |
| *Listeria monocytogenes* | 10-092876-1235 LM8 | GCA_001999085.1 | 2.96682 | 37.8 | 1 |
| *Listeria monocytogenes* | 10-092876-0145 LM9 | GCA_001999105.1 | 3.07003 | 38 | 1 |
| *Listeria monocytogenes* | 10-092876-1763 LM10 | GCA_001999125.1 | 2.94091 | 37.9 | 1 |
| *Listeria monocytogenes* | 10-092876-1016 LM11 | GCA_001999145.1 | 2.96566 | 38 | 1 |
| *Listeria monocytogenes* | 10-092876-0769 LM12 | GCA_001999165.1 | 2.92963 | 38 | 1 |
| *Listeria monocytogenes* | PNUSAL000144 | GCA_002105675.1 | 2.9122 | 38 | 1 |
| *Listeria monocytogenes* | CFSAN004330 | GCA_002105715.1 | 3.05793 | 37.89 | 2 |
| *Listeria monocytogenes* | HPB2863_1 | GCA_002144215.1 | 2.84753 | 37.9 | 10 |
| *Listeria monocytogenes* | HPB1631_1 | GCA_002144235.1 | 2.843 | 38.1 | 11 |
| *Listeria monocytogenes* | HPB1631_100 | GCA_002144255.1 | 2.84373 | 38.1 | 12 |
| *Listeria monocytogenes* | 02-6679 | GCA_002213505.1 | 2.908 | 38 | 1 |
| *Listeria monocytogenes* | 01-5252 | GCA_002213525.1 | 2.97444 | 37.9 | 1 |
| *Listeria monocytogenes* | 02-5993 | GCA_002213545.1 | 2.99925 | 38 | 1 |
| *Listeria monocytogenes* | 04-5457 | GCA_002213565.1 | 2.99933 | 38 | 1 |
| *Listeria monocytogenes* | 08-6056 | GCA_002213585.1 | 3.03262 | 38 | 1 |
| *Listeria monocytogenes* | 08-7374 | GCA_002213605.1 | 2.99933 | 38 | 1 |
| *Listeria monocytogenes* | 08-7669 | GCA_002213625.1 | 2.95458 | 38 | 1 |
| *Listeria monocytogenes* | 10-1046 | GCA_002213645.1 | 2.99821 | 38 | 1 |
| *Listeria monocytogenes* | 10-1321 | GCA_002213665.1 | 2.99933 | 38 | 1 |
| *Listeria monocytogenes* | 95-0093 | GCA_002213685.1 | 3.00027 | 38 | 1 |
| *Listeria monocytogenes* | 98-2035 | GCA_002213705.1 | 3.03221 | 38 | 1 |
| *Listeria monocytogenes* | 99-6370 | GCA_002213725.1 | 3.03221 | 38 | 1 |
| *Listeria monocytogenes* | 10-5025 | GCA_002213745.1 | 2.96752 | 37.9 | 1 |
| *Listeria monocytogenes* | 10-5026 | GCA_002213765.1 | 2.9675 | 37.9 | 1 |
| *Listeria monocytogenes* | 10-5027 | GCA_002213785.1 | 2.96533 | 37.9 | 1 |
| *Listeria monocytogenes* | 10-4754 | GCA_002213805.1 | 2.91092 | 38 | 1 |
| *Listeria monocytogenes* | 10-4758 | GCA_002213825.1 | 2.91092 | 38 | 1 |
| *Listeria monocytogenes* | 10-0933 | GCA_002213845.1 | 2.87028 | 38 | 1 |
| *Listeria monocytogenes* | 10-0934 | GCA_002213865.1 | 2.87028 | 38 | 1 |
| *Listeria monocytogenes* | 02-1103 | GCA_002213885.1 | 2.97911 | 37.9 | 1 |
| *Listeria monocytogenes* | 10-5024 | GCA_002213905.1 | 2.99933 | 38 | 1 |
| *Listeria monocytogenes* | 02-1289 | GCA_002213925.1 | 2.97911 | 37.9 | 1 |
| *Listeria monocytogenes* | 02-1792 | GCA_002213945.1 | 2.97911 | 37.9 | 1 |
| *Listeria monocytogenes* | 02-6680 | GCA_002213965.1 | 2.908 | 38 | 1 |
| *Listeria monocytogenes* | 81-0558 | GCA_002213985.1 | 2.97154 | 38 | 1 |
| *Listeria monocytogenes* | 81-0592 | GCA_002214005.1 | 2.97586 | 38 | 1 |
| *Listeria monocytogenes* | 01-1468 | GCA_002214025.1 | 3.02176 | 37.9 | 1 |
| *Listeria monocytogenes* | 10-0814 | GCA_002214045.1 | 2.99931 | 38 | 1 |
| *Listeria monocytogenes* | 10-0809 | GCA_002214065.1 | 2.97585 | 38 | 1 |
| *Listeria monocytogenes* | 10-0810 | GCA_002214085.1 | 3.01794 | 38 | 1 |
| *Listeria monocytogenes* | 10-0811 | GCA_002214105.1 | 3.01794 | 38 | 1 |
| *Listeria monocytogenes* | 10-0812 | GCA_002214125.1 | 2.94223 | 38 | 1 |
| *Listeria monocytogenes* | 10-0813 | GCA_002214145.1 | 2.94223 | 38 | 1 |
| *Listeria monocytogenes* | FORC_049 | GCA_002220325.1 | 2.93503 | 38 | 1 |
| *Listeria monocytogenes* | 01-6771 | GCA_002240245.1 | 2.93018 | 37.9 | 1 |
| *Listeria monocytogenes* | 03-5473 | GCA_002240265.1 | 2.9743 | 37.9 | 1 |
| *Listeria monocytogenes* | ATCC 7644 | GCA_002250305.1 | 2.96428 | 37.8 | 11 |
| *Listeria monocytogenes* | CFSAN049319 | GCA_002443355.1 | 2.91495 | 37.9 | 30 |
| *Listeria monocytogenes* | CFSAN049229 | GCA_002444445.1 | 3.07776 | 37.8 | 38 |
| *Listeria monocytogenes* | CFSAN049284 | GCA_002464905.1 | 2.93899 | 37.8 | 21 |
| *Listeria monocytogenes* | AT3E | GCA_002557735.1 | 3.11633 | 37.9737 | 2 |
| *Listeria monocytogenes* | AL4E | GCA_002557815.1 | 3.028 | 38 | 1 |
| *Listeria monocytogenes* | NH1 | GCA_002969195.1 | 3.00249 | 37.9 | 1 |
| *L. innocua* | Clip11262 | GCA_000195795.1 | 3.09311 | 37.3497 | 2 |
| *L. innocua* | FSL S4-378 | GCA_000183885.1 | 3.11594 | 37.4519 | 2 |
| *L. innocua* | [ATCC 33091](https://www.ncbi.nlm.nih.gov/genome/1024?genome_assembly_id=1470399#!/prokaryotes/1024/_blank) | GCA_000241405.1 | 2.89 | 37.40 | 17 |
| *L. innocua* | [MOD1_LS888](https://www.ncbi.nlm.nih.gov/genome/1024?genome_assembly_id=212471#!/prokaryotes/1024/_blank) | GCA_000773035.1 | 2.89091 | 37.3 | 12 |
| *L. innocua* | [9KSM](https://www.ncbi.nlm.nih.gov/genome/1024?genome_assembly_id=228656#!/prokaryotes/1024/_blank) | GCA_000960585.1 | 2.91116 | 37.4 | 21 |
| *L. innocua* | [12KSM](https://www.ncbi.nlm.nih.gov/genome/1024?genome_assembly_id=228657#!/prokaryotes/1024/_blank) | GCA_000960735.1 | 2.91057 | 37.3 | 22 |
| *L. ivanovii* | FSL F6-596 | GCA_000183925.1 | 3.13339 | 35.7137 | 1 |
| *L. ivanovii* | PAM 55 | GCA_000252975.1 | 2.92888 | 37.1 | 1 |
| *L. ivanovii* | WSLC3009 | GCA_000565155.1 | 2.91954 | 37.2 | 1 |
| *L. ivanovii* | WSLC 30167 | GCA_000763475.1 | 2.99349 | 37 | 1 |
| *L. ivanovii* | WSLC 30151 | GCA_000763495.1 | 3.04503 | 37.1 | 1 |
| *L. ivanovii* | WSLC 3010 | GCA_000763515.1 | 2.91955 | 37.2 | 1 |
| *L. ivanovii* | G_770 | GCA_001049935.1 | 2.96554 | 37.1 | 17 |
| *L. ivanovii* | ATCC 49954 | GCA_900106685.1 | 2.9647 | 36.9 | 33 |
| *L. ivanovii* | NCTC11846 | GCA_900187025.1 | 2.91955 | 37.2 | 1 |
| *L. seeligeri* | SLCC3954 | GCA_000027145.1 | 2.79764 | 37.4 | 1 |
| *L. seeligeri* | FSL N1-067 | GCA_000183945.1 | 3.14016 | 37.3 | 1 |
| *L. seeligeri* | FSL S4-171 | GCA_000183965.1 | 2.97234 | 37.2514 | 1 |
| *L. seeligeri* | BCW_4759 | GCA_001939275.1 | 2.89781 | 37.3 | 19 |
| *L. welshimeri* | SLCC5334 | GCA_000060285.1 | 2.81413 | 36.4 | 1 |
| *L. welshimeri* | NCTC11857 | GCA_900187315.1 | 2.81414 | 36.4 | 1 |
| *L. grayi* | DSM 20601 | GCA_000148995.1 | 2.59832 | 41.6 | 4 |
| *L. grayi* | FSL F6-1183 | GCA_000525895.1 | 2.71911 | 41.4 | 35 |
| *L. fleischmanii* | 1991 | GCA_001050295.1 | 2.79274 | 38.4 | 17 |
| *L. fleischmanii* | LU2006-1 | GCA_000344175.1 | 2.84113 | 38.3 | 102 |
| *L. fleischmanii* | FSL S10-1203 | GCA_000526015.1 | 3.18779 | 38.4 | 126 |
| *L. fleischmanii* | TTU M1-001 | GCA_000252625.2 | 2.81751 | 38.6 | 406 |
| *L. newyorkensis* | FSL M6-0635 | GCA_000766145.1 | 3.51544 | 42.9 | 47 |
| *L. newyorkensis* | 1903 | GCA_001050305.1 | 3.45309 | 43.1 | 79 |
| *L. newyorkensis* | F1604011-044 | GCA_002894185.1 | 3.54036 | 42.8 | 50 |
| *L. aquatica* | FSL S10-1188 | GCA_000525795.1 | 2.60115 | 39.5 | 31 |
| *L. booriae* | FSL A5-0281 | GCA_000766865.1 | 3.43696 | 42.1 | 31 |
| *L. cornellensis* | FSL F6-0969 | GCA_000525855.1 | 3.34783 | 41.9 | 91 |
| *L. floridensis* | FSL S10-1187 | GCA_000525875.1 | 2.79439 | 41 | 92 |
| *L. grandensis* | FSL F6-0971 | GCA_000525835.1 | 3.18173 | 42 | 62 |
| *L. riparia* | FSL S10-1204 | GCA_000525995.1 | 3.29104 | 41 | 69 |
| *L. rocourtiae* | FSL F6-920 | GCA_000525975.1 | 3.21675 | 40.3 | 126 |
| *Escherichia coli* | K-12 substr. MG1655 | GCA_000005845.2 | 4.64165 | 50.8 | 1 |
| *Escherichia coli* | CFT073 | GCA_000007445.1 | 5.23143 | 50.5 | 1 |
| *Escherichia coli* | Sakai substr. RIMD 0509952 | GCA_000008865.2 | 5.5946 | 50.4477 | 3 |
| *Escherichia coli* | BL21(DE3) | GCA_000009565.2 | 4.55895 | 50.8 | 1 |
| *Escherichia coli* | K-12 substr. W3110 | GCA_000010245.1 | 4.64633 | 50.8 | 1 |
| *Salmonella enterica* | LT2 | GCA_000006945.2 | 4.95138 | 52.2171 | 2 |
| *Salmonella enterica* | Ty2 | GCA_000007545.1 | 4.79196 | 52.1 | 1 |
| *Salmonella enterica* | SC-B67 | GCA_000008105.1 | 4.944 | 52.1737 | 3 |
| *Salmonella enterica* | P125109 | GCA_000009505.1 | 4.68585 | 52.2 | 1 |
| *Salmonella enterica* | 287/91 | GCA_000009525.1 | 4.6587 | 52.2 | 1 |
| *Bacillus cereus* | ATCC 14579 | GCA_000007825.1 | 5.42708 | 35.3076 | 2 |
| *Bacillus cereus* | ATCC 10987 | GCA_000008005.1 | 5.43265 | 35.5156 | 2 |
| *Bacillus cereus* | E33L | GCA_000011625.1 | 5.84323 | 35.1687 | 6 |
| *Bacillus cereus* | Q1 | GCA_000013065.1 | 5.50621 | 35.504 | 3 |
| *Bacillus cereus* | B4264 | GCA_000021205.1 | 5.41904 | 35.3 | 1 |
| *Campylobacter jejuni* | NCTC 11168 | GCA_000009085.1 | 1.64148 | 30.5 | 1 |
| *Campylobacter jejuni* | RM1221 | GCA_000011865.1 | 1.77783 | 30.3 | 1 |
| *Campylobacter jejuni* | 81-176 | GCA_000015525.1 | 1.69905 | 30.4566 | 3 |
| *Campylobacter jejuni* | 269.97 | GCA_000017485.1 | 1.84511 | 30.6 | 1 |
| *Campylobacter jejuni* | 81116; NCTC 11828 | GCA_000017905.1 | 1.62811 | 30.5 | 1 |
| *Staphylococcus* haemolyticus | JCSC1435 | GCA_000009865.1 | 2.69786 | 32.7885 | 4 |
| Staphylococcus haemolyticus | Sh29/312/L2 | GCA_000972725.1 | 2.56137 | 32.7 | 1 |
| Staphylococcus haemolyticus | 109_SHAE | GCA_001068645.1 | 7.08819 | 44.4 | 516 |
| Staphylococcus haemolyticus | 1328_SHAE | GCA_001069705.1 | 2.58705 | 32.7 | 147 |
| Staphylococcus haemolyticus | 1292_SHAE | GCA_001070475.1 | 2.59602 | 32.8 | 124 |
| *Shigella sonnei* | Ss046 | GCA_000092525.1 | 5.05532 | 50.7587 | 5 |
| *Shigella sonnei* | 53G | GCA_000283715.1 | 5.22047 | 50.7341 | 5 |
| *Shigella sonnei* | FORC_011 | GCA_001518855.1 | 5.13271 | 50.7531 | 4 |
| *Shigella sonnei* | FDAARGOS_90 | GCA_001558295.2 | 4.98834 | 51.0021 | 6 |
| *Shigella sonnei* | 2015AM-1099 | GCA_002142635.1 | 4.93557 | 51 | 1 |
| *Bacillus subtilis* | 168 | GCA_000009045.1 | 4.21561 | 43.5 | 1 |
| *Bacillus subtilis* | W23 | GCA_000146565.1 | 4.02768 | 43.9 | 1 |
| *Bacillus subtilis* | BSn5 | GCA_000186745.1 | 4.0936 | 43.8 | 1 |
| *Bacillus subtilis* | BEST195 | GCA_000209795.2 | 4.11122 | 43.4962 | 2 |
| *Bacillus subtilis* | TU-B-10 | GCA_000227465.1 | 4.20722 | 43.8 | 1 |
| *Vibrio parahaemolyticus* | O3:K6 substr. RIMD 2210633 | GCA_000196095.1 | 5.16577 | 45.4 | 2 |
| *Vibrio parahaemolyticus* | BB22OP | GCA_000328405.1 | 5.10352 | 45.3292 | 2 |
| *Vibrio parahaemolyticus* | FDA_R31 | GCA_000430405.1 | 5.22356 | 45.3287 | 2 |
| *Vibrio parahaemolyticus* | CDC_K4557 | GCA_000430425.1 | 5.13858 | 45.3352 | 2 |
| *Vibrio parahaemolyticus* | UCM-V493 | GCA_000568495.1 | 5.23257 | 45.3212 | 3 |
