# Supplementary material for: Multiplex PCR for the Identification of Pathogenic Listeria in Flammulina velutipes Plant Based on Novel Specific Targets Revealed by Pan-Genome Analysis
Source: Front Microbiol. 2021 Jan 15;11:634255. doi: 10.3389/fmicb.2020.634255 (PMC7843925; doi:10.3389/fmicb.2020.634255)
Supplement: Supplementary file 2 [file Data_Sheet_2.doc]

**Supplementary Material (2)**

**Multiplex PCR for the Identification of Pathogenic *Listeria* in** ***Flammulina velutipes* Plant** **Based on Novel Specific Targets Revealed by Pan-genome Analysis**

Fan Li ^1, 2 #^, Qinghua Ye ^1 #^, Moutong Chen ^1, *^, Jumei Zhang ^1^, Liang Xue ^1^, Juan Wang ^3^, Shi Wu ^1^, Haiyan Zeng ^1^, Qihui Gu ^1^, Youxiong Zhang ^1^, Xianhu Wei ^1^, Yu Ding ^4 *^, Qingping Wu ^1 *^

^1^ Guangdong Provincial Key Laboratory of Microbial Safety and Health, State Key Laboratory of Applied Microbiology Southern China, Guangdong Institute of Microbiology, Guangdong Academy of Sciences, Guangzhou, China

^2^ School of Biology and Biological Engineering, South China University of Technology, Guangzhou, China

^3^ College of Food Science, South China Agricultural University, Guangzhou, China

^4^ Department of Food Science and Technology, Jinan University, Guangzhou, China

**^*^ Corresponding author:**

Qingping Wu

1. mail: [wuqp203@163.com](mailto:wuqp203@163.com)

Tel: +86-20-87688132; fax: +86-20-87688132

Address: Guangdong Institute of Microbiology, Yard 100#, Xianlie Zhong Lu, Yuexiu District, 510070 Guangzhou, P.R.China

Yu Ding

E-mail: dingyu@jnu.edu.cn

Tel: +86-20-85222379

Address: Department of Food Science & Technology, Institute of Food Safety and Nutrition, Jinan University, Huangpu Ave. 601, Guangzhou 510632, China

^#^ Fan Li, Qinghua Ye, and Moutong Chen contribute to the manuscript equally.

**Table S2.** *Listeria* strains and other common foodborne pathogenic strains tested in this study and mPCR results of specificity tests.

| **No.** | **Bacterial species** | **Serotype** | **Strain** | **Number of strains** | **Source*** | **mPCR primer paris** | | |
| --- | --- | --- | --- | --- | --- | --- | --- | --- |
|  |  |  |  |  |  | **SPP1** | **LM1** | **LIV2** |
| 1 | *L. monocytogenes* | 1/2a | 1646-2LM | 1 | a | + | + | - |
| 2 | *L. monocytogenes* | 3a | ^1^ATCC 51782 | 1 | b | + | + | - |
| 3 | *L. monocytogenes* | 1/2c |  | 1 | a | + | + | - |
| 4 | *L. monocytogenes* | 4b | ATCC 19115 | 1 | d | + | + | - |
| 5 | *L. monocytogenes* | 4d | ATCC 19117 | 1 | c | + | + | - |
| 6 | *L. monocytogenes* | 4e | ATCC 19118 | 1 | c | + | + | - |
| 7 | *L. monocytogenes* | 4ab | Murray B | 1 | c | + | + | - |
| 8 | *L. monocytogenes* | 1/2b |  | 1 | a | + | + | - |
| 9 | *L. monocytogenes* | 3b |  | 1 | c | + | + | - |
| 10 | *L. monocytogenes* | 7 | ^3^SLCC 2428 | 1 | c | + | + | - |
| 11 | *L. monocytogenes* | 4a | ATCC 19114 | 1 | c | + | + | - |
| 12 | *L. monocytogenes* | 4c |  | 1 | a | + | + | - |
| 13 | *L. ivanovii* | 5 | ATCC 19119 | 1 | e | + | - | + |
| 14 | *L. innocua* | 6a | ATCC 33090 | 1 | d | + | - | - |
| 15 | *L. seeligeri* | 1/2b | ^4^CICC 21671 | 1 | c | + | - | - |
| 16 | *L. welshimeri* | 6b | ATCC 35897 | 1 | e | + | - | - |
| 17 | *L. grayi* |  | ATCC 19120 | 1 | d | + | - | - |
| 18 | *Cronobacter sakazakii* |  | ATCC 29544 | 1 | d | - | - | - |
| 19 | *Cronobacter sakazakii* |  |  | 1 | a | - | - | - |
| 20 | *Staphylococcus aureus* |  | ATCC 25923 | 1 | d | - | - | - |
| 21 | *Staphylococcus aureus* |  | ATCC 29213 | 1 | d | - | - | - |
| 22 | *Pseudomonas aeruginosa* |  | ATCC 9027 | 1 | d | - | - | - |
| 23 | *Pseudomonas aeruginosa* |  | ATCC 27853 | 1 | d | - | - | - |
| 24 | *Salmonella Enteritidis* |  | CMCC 50335 | 1 | d | - | - | - |
| 25 | *Salmonella Typhimurium* |  | ATCC 14028 | 1 | d | - | - | - |
| 26 | *Campylobacter jejuni* |  | ATCC 33291 | 1 | d | - | - | - |
| 27 | *Escherichia coli* |  | ATCC 25922 | 1 | d | - | - | - |
| 28 | *Shigella sonnei* |  | CMCC(B) 51592 | 1 | d | - | - | - |
| 29 | *Bacillus cereus* |  | ATCC 14579 | 1 | a | - | - | - |
| 30 | *Yersinia enterocolitica* |  |  | 1 | d | - | - | - |
| 31 | *Yersinia enterocolitica* |  |  | 1 | d | - | - | - |
| 32 | *Vibrio parahemolyticus* |  | ATCC 33847 | 1 | d | - | - | - |
| 33 | *Vibrio parahemolyticus* |  | ATCC 17802 | 1 | d | - | - | - |

^*^ a, our laboratory; b, Chinese Center for Disease Control and Prevention, China; c, College of Food Science and Technology, Nanjing Agricultural University, China ; d, Guangdong Huankai Co., Ltd., China; e, State Key Laboratory of Food Science and Technology, Nanchang University, China.

^1^ ATCC, American Type Culture Collection, USA.

^2^ CMCC, China Medical Culture Collection, China.

^3^ SLCC, Seeliger’s Special Listeria Culture Collection, Germany.

^4^ CICC, Center of Industrial Culture Collection, China.

Result (+/-) indicate positive and negative signals.

**Table S3.** PCR detection sensitivity of each target genes specific for *Listeria* species.

| **Species** | **Gene location*** | **Gene** | **Sensitivity (CFU/mL)** | **Product size (bp)** | **Applied to mPCR system** |
| --- | --- | --- | --- | --- | --- |
| *Listeria* spp. | 979606 - 980076 | *LMO**SLCC2755_0944* | 10^3^ | 427 | yes |
| *L. monocytogenes* | 91271 - 91639 | *LMOSLCC2755_0090* | 10^3^ | 260 | yes |
| *L. ivanovii* | 1124254 - 1124736 | *NCTC12701_01099* | 10^4^ | 264 |  |
|  | 418244 - 418759 | *queT_1* | 10^3^ | 144 | yes |
|  | 848588 - 849271 | *gmuC_3* | 10^3^ | 452 |  |

* Reference strain are *L. monocytogenes* str. SLCC2755, *L. ivanovii* subsp. londoniensis str. NCTC12701.

Table S4. Detection results of pathogenic *Listeria* in artificial *Flammulina velutipes* by multiplex PCR.

| Initial inoculum (CFU/10g *F. velutipes*) | Multiplex PCR results of different enrichment time | | | | |
| --- | --- | --- | --- | --- | --- |
|  | 4 h | 6 h | 8 h | 10 h | 12 h |
| 7.6 × 10^4^ | + | + | + | + | + |
| 7.6 × 10^3^ | - | + | + | + | + |
| 7.6 × 10^2^ | - | - | + | + | + |
| 7.6 × 10^1^ | - | - | - | + | + |
| 7.6 × 10^0^ | - | - | - | - | + |

Table S5. Natural *Flammulina velutipes* plants samples tests using multiplex PCR.

| Sample sites | Sample types | Number of samples | Number of positive result in mPCR | | |
| --- | --- | --- | --- | --- | --- |
|  |  |  | *Listeria spp.* | *L. monocytogenes* | *L. ivanovii* |
| Composting phase (n=15) | compost | 9 | 2 | 0 | 0 |
|  | sterile compost | 6 | 0 | 0 | 0 |
| Mycelium culture room (n=18) | cultures | 9 | 1 | 0 | 0 |
|  | shelf surfaces | 9 | 0 | 0 | 0 |
| Mycelium stimulation room (n=18) | mycelium stimulation machinery | 9 | 9 | 9 | 0 |
|  | floor | 9 | 9 | 8 | 0 |
| Fruiting body cultivation room (n=27) | drains | 9 | 7 | 6 | 0 |
|  | shelf surfaces | 9 | 4 | 3 | 0 |
|  | *Flammulina velutipes* | 9 | 3 | 1 | 0 |
| Harvesting room (n=51) | package machinery surfaces | 3 | 0 | 0 | 0 |
|  | scales | 6 | 3 | 0 | 0 |
|  | conveyor belts | 9 | 9 | 6 | 0 |
|  | cutler surfaces | 9 | 8 | 3 | 0 |
|  | packaged *Flammulina velutipes* | 6 | 5 | 3 | 0 |
|  | floor | 6 | 5 | 5 | 0 |
|  | drains | 6 | 6 | 6 | 0 |
|  | workers | 6 | 4 | 3 | 0 |

**Figure S1**


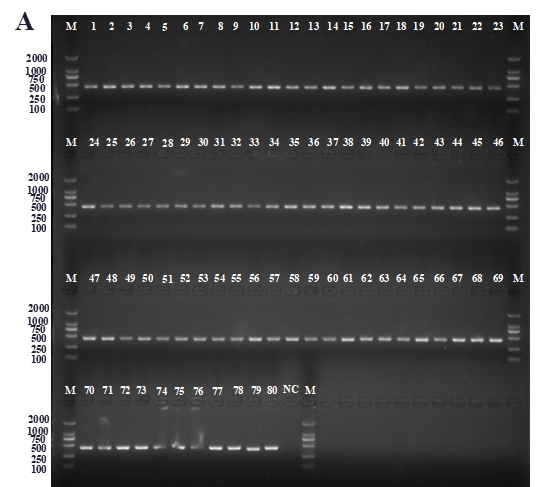


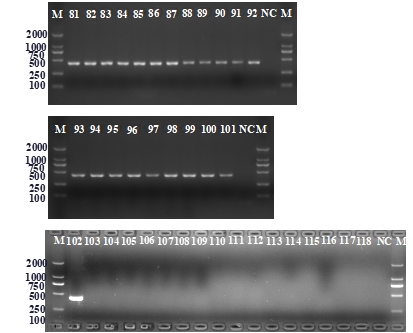


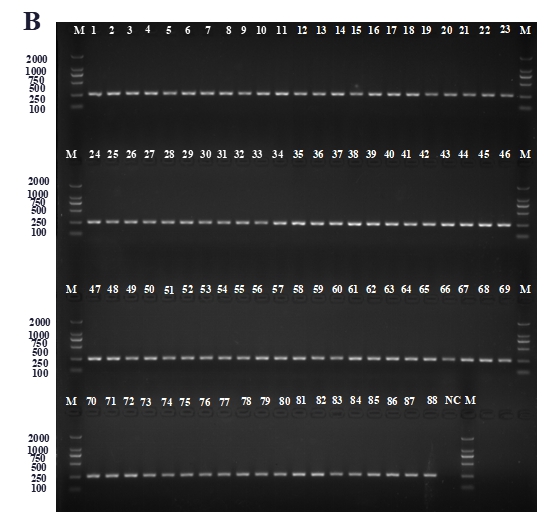


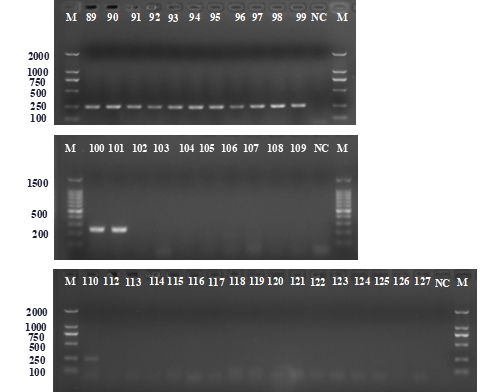


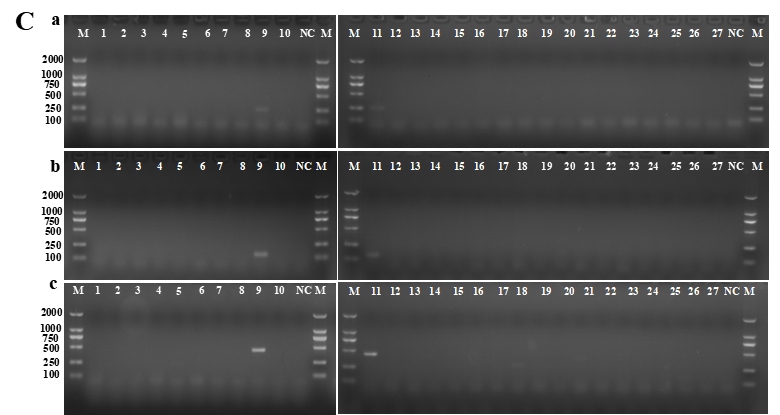


**Figure S1.** Screening of targets for the genera and species of *Listeria* by end-point PCR.

1. PCR results of novel molecular detection gene (*LMOSLCC2755_0944*) specific for the *Listeria* spp. Lane M: DL2000 DNA standard marker; lanes 1-93: the strains of different serotypes of *L. monocytogenes*; lanes 94-102: six common *Listeria* strains; lanes 103-118: non-*Listeria* strains; Lane NC: negative control.
2. PCR results of novel molecular detection gene (*LMOSLCC2755_0090*) specific for *L. monocytogenes*. Lane M: DL2000 DNA standard marker; lanes 1-101 and 110: different serotype strains of *L. monocytogenes*; lanes 102-109: other five common *Listeria* strains; lanes 112-127: non- *Listeria* strains; lane NC: negative control.
3. PCR results of novel molecular detection genes (*NCTC12701_01099* (a), *queT_1* (b), and *gmuC_3* (c)) specific for *L. ivanovii*. Lane M: DL2000 DNA standard marker; lanes 9 and 11: *L. ivanovii* strains; lanes 1-8 and 10: other common *Listeria* strains; lanes 12-27: non-*Listeria* strains; lane NC: negative control.
